# Endocytosis is required for consolidation of pattern-separated memories in the perirhinal cortex

**DOI:** 10.3389/fnsys.2023.1043664

**Published:** 2023-02-23

**Authors:** Dinka Piromalli Girado, Magdalena Miranda, Marcelo Giachero, Noelia Weisstaub, Pedro Bekinschtein

**Affiliations:** Laboratory of Memory and Molecular Cognition, Instituto de Neurociencia Cognitiva y Traslacional (Consejo Nacional de Investigaciones Científicas y Técnicas–Fundación INECO–Universidad Favaloro), Buenos Aires, Argentina

**Keywords:** pattern separation, object recognition, perirhinal cortex, brain derived neurotrophic factor (BDNF), endocytosis

## Abstract

**Introduction:**

The ability to separate similar experiences into differentiated representations is proposed to be based on a computational process called pattern separation, and it is one of the key characteristics of episodic memory. Although pattern separation has been mainly studied in the dentate gyrus of the hippocampus, this cognitive function if thought to take place also in other regions of the brain. The perirhinal cortex is important for the acquisition and storage of object memories, and in particular for object memory differentiation. The present study was devoted to investigating the importance of the cellular mechanism of endocytosis for object memory differentiation in the perirhinal cortex and its association with brain-derived neurotrophic factor, which was previously shown to be critical for the pattern separation mechanism in this structure.

**Methods:**

We used a modified version of the object recognition memory task and intracerebral delivery of a peptide (Tat-P4) into the perirhinal cortex to block endocytosis.

**Results:**

We found that endocytosis is necessary for pattern separation in the perirhinal cortex. We also provide evidence from a molecular disconnection experiment that BDNF and endocytosis-related mechanisms interact for memory discrimination in both male and female rats.

**Discussion:**

Our experiments suggest that BDNF and endocytosis are essential for consolidation of separate object memories and a part of a time-restricted, protein synthesis-dependent mechanism of memory stabilization in Prh during storage of object representations.

## 1. Introduction

Consolidation of similar experiences as of distinct representations is a key factor for an accurate retrieval of episodic memories (Dickerson and Eichenbaum, 2010). This ability to separate similar memories into unique representations is thought to rely on pattern separation, a process of orthogonalization that has been postulated using computational models (Marr, 1971; Treves and Rolls, 1994; McClelland et al., 1995; Rolls and Kesner, 2006). There is electrophysiological evidence that the dentate gyrus (DG) and the perirhinal cortex (Prh) are critical regions in the control of this phenomenon (Leutgeb et al., 2007; Neunuebel and Knierim, 2014; Ahn and Lee, 2017). Since episodic memory involves the recollection of unique events, separation of similar experiences is proposed to be a key component for the storage of non-confusable representations of similar experiences, especially in the hippocampus (Ranganath, 2010). Nevertheless, it has been pointed out that Prh could be an important structure involved in the consolidation of object recognition memory and where pattern separation could also occur (Zhu et al., 1995; Winters et al., 2004; Bartko et al., 2007; Miranda et al., 2017, 2020; Miranda and Bekinschtein, 2018).

Endocytosis is a fundamental process for neuronal function controlling the recycle of presynaptic vesicles, as well as the trafficking of plasma membrane receptors, ion channels, and transporters (Parton and Dotti, 1993; Haucke and Klingauf, 2007; Rosendale et al., 2017). Impairments in the endocytic pathway have been associated with the pathophysiology of certain neurological diseases such as amyotrophic lateral sclerosis, Alzheimer’s disease, and Parkinson’s disease (Parton and Dotti, 1993). Several studies have shown the critical role of receptor endocytosis during consolidation in different memory tasks (Abe et al., 2004; Winters and Bussey, 2005a; Dalton et al., 2008; Wang et al., 2017; Awasthi et al., 2019). Some of these receptors are critical for memory processes. Blockade of either NMDA or AMPA receptor using a broad-spectrum glutamate receptor antagonist within the anteromedial portion of Prh is sufficient to disrupt object recognition memory in macaques (Malkova et al., 2015). Interestingly, blocking the endocytosis of AMPA receptors in rat Prh prior to the retrieval phase with an interference peptide disrupted object recognition memory (Cazakoff and Howland, 2011). Nevertheless, how endocytosis interacts with other plasticity molecules to support memory, has not been studied.

Regarding the molecular mechanisms underlying memory storage, BDNF is a pivotal neurotrophin for learning and memory, including object recognition memory (Bekinschtein et al., 2014; Miranda et al., 2017). Previous studies showed BDNF mediates molecular mechanisms that are essential for the consolidation of similar and dissimilar spatial and object memories in the DG (Bekinschtein et al., 2013) and the Prh (Miranda et al., 2017, 2020). BDNF knockdown impairs long-term memory consolidation in the Prh (Seoane et al., 2011). Moreover, our group has shown that rats separate memories of ambiguous information engaging in a BDNF-associated process specifically in the Prh (Miranda et al., 2017, 2020). BDNF has a strong interaction with different types of receptors, such as glutamate receptors (AMPAr and NMDAr) and GABA receptors (GABAr) (Carvalho et al., 2008; Kealy and Commins, 2009; Lu et al., 2015; Saffarpour et al., 2017; Miranda and Bekinschtein, 2018). Phosphorylation potentiates NMDA currents in hippocampus, and it has been proposed that BDNF phosphorylation modulates NMDA receptors, enhancing synaptic transmission and playing a role in long term potentiation (Suen et al., 1997; Lin et al., 1998). In some diseases in which pattern separation is compromised, like schizophrenia, there was a clear dysregulation of AMPAr levels and BDNF signaling (Kennedy et al., 2003; Nawa and Takei, 2006; Watanabe et al., 2010; Zeppillo et al., 2020), although the functional consequence of these in this specific pathology is not yet clear. It has also been established that BDNF activates, through TrkB, a process that leads to a rapid decrease in the GABA-A receptor in the postsynaptic membrane, modulating GABA receptors trafficking (Brünig et al., 2001; Cheng and Yeh, 2003).

This study focused on the role of endocytosis in Prh and how it interacts with BDNF during consolidation of similar object memories. Since activation of the BDNF-TrkB pathway can lead to receptor endocytosis and modify synaptic plasticity, we wondered if endocytosis could be a potential molecular mechanism involved in mnemonic differentiation of objects in the Prh. Thus, this study explores a potential BDNF-dependent intracellular mechanism for discrimination of similar, but not dissimilar objects. This set of results advances further in the understanding of the molecular mechanisms of memory storage in the Prh we have been studying for many years (Miranda et al., 2017, 2020). We used a dynamin function-blocking peptide (Tat-P4) to block Prh endocytosis and found that endocytosis is necessary for consolidation of similar, but not dissimilar object memories. In addition, we provide evidence that BDNF could be interacting with pathways of endocytosis to exert its effects.

## 2. Materials and methods

### 2.1. Subjects

**FIGURE 1:**
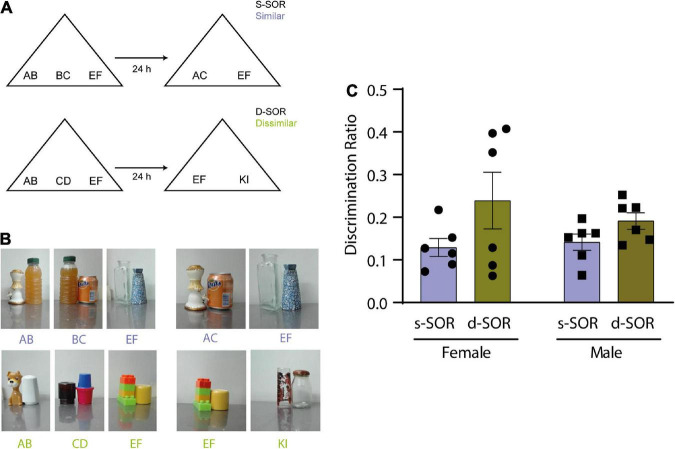
**(A)** Diagram of the spontaneous object recognition task (SOR) in the similar version (s-SOR) (top) and the dissimilar version (d-SOR) (up). **(B)** Examples of a set of objects used in each condition (bottom). **(C)** Discrimination ratios during the choice phase 24 h after sample phase, in the s-SOR and d-SOR condition in female and male rats. Repeated-measures two-way ANOVA; *F* = 3.741, pcondition = 0.0819, *F* = 0.2857, psex = 0.6047, *F* = 0.5327, pinteraction = 0.4822.

**FIGURE 2:**
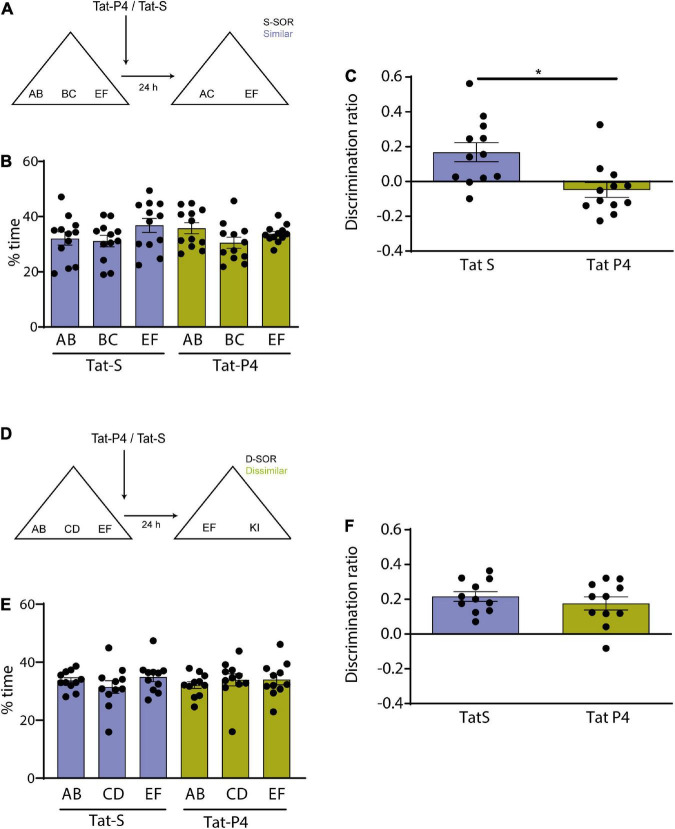
**(A)** Schematic illustration of the similar-spontaneous object recognition task (s-SOR) task indicating when Tat-P4/Tat-S was infused. **(B)** Percentage of time spent exploring each of the objects in the sample phase in the s-SOR. Repeated-measures two-way ANOVA (%time); *F* = 1.000, pdrug = 0.3388, *F* = 2.045, pobject = 0.1533, *F* = 0.7906, pinteraction = 0.4660. **(C)** Effect of Tat-P4 or Tat-S injections on the discrimination ratios for the s-SOR version of the task. Paired *t*-test (*t* = 2.899), *p* = 0.0145, *n* = 12. **(D)** Schematic illustration of the dissimilar (d-SOR) task indicating when Tat-P4/Tat-S was infused. **(E)** Percentage of time spent exploring each of the objects in the sample phase in the d-SOR. Repeated-measures two-way ANOVA (%time); *F* = 0.1020, pdrug = 0.7560, *F* = 0.4530, pobject = 0.6421, *F* = 0.5049, pinteraction = 0.6111. **(F)** Effect of Tat-P4 or Tat-S injections on the discrimination ratios for the d-SOR version of the task. Paired *t*-test (*t* = 0.8728), *p* = 0.4033, *n* = 11. *p < 0.05.

**FIGURE 3:**
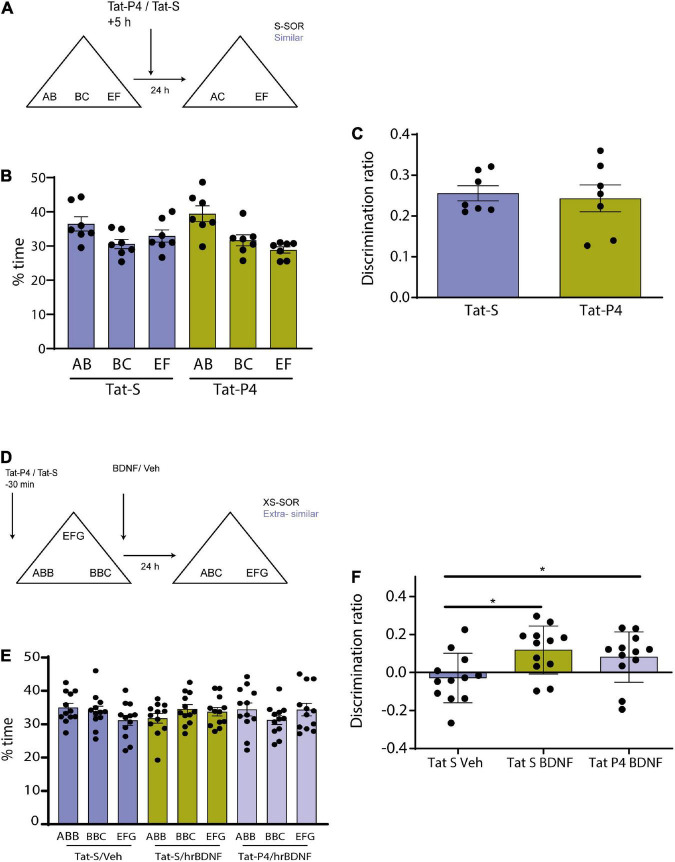
**(A)** Schematic illustration of the similar-spontaneous object recognition task (s-SOR) task indicating infusion of Tat-P4/Tat-S 5 hs after the sample phase. **(B)** Percentage of time spent exploring each of the objects in the sample phase. Repeated-measures two-way ANOVA (%time) *F* = 2.400, pdrug = 0.1723, *F* = 6.509, pobject = 0.0122, *F* = 01.608, pinteraction = 0.2407. **(C)** No memory impairment found in animals between conditions [Tat-S vs. Tat-P4 paired *t*-test (*t* = 0.2985), *p* = 0.7754, *n* = 7]. Both animal groups learned the task [One-sample *t*-test (Tat-S, *t* = 13.88), *p* < 0.0001; one- sample *t*-test (Tat-P4, *t* = 7.377), *p* = 0.0003]. **(D)** Schematic illustration of the extra similar condition of the task (xs-SOR) indicating the time points at which Tat-P4 or Tat-S and hrBDNF or vehicle were infused 30 min before sample phase and immediately after sample phase, respectively. **(E)** Percentage of time spent exploring each of the objects in the sample phase. Repeated-measures two-way ANOVA (%time), *F* = 1.000, pdrug = 0.1748, *F* = 0.1089, pobject = 0.8668, *F* = 1.191, pinteraction = 0.3282. **(F)** Repeated measures one-way ANOVA (%time), *F* = 3.838, pdrug = 0.0430, *F* = 0.7578, pindividual = 0.6757. *p < 0.05.

The subjects were 83 Long-Evans rats from our breeding colony, of which 52 were female and 31 males, that conformed mixed groups in some experiments (Figures 1, 2, 3B, C). The subjects weighed 200–300 g at the start of testing. The rats were housed on a reversed 12-h light/12-h dark cycle (lights on 19:00–07:00), in groups of two or four. All behavioral testing was conducted during the dark phase of the cycle. Rats were food deprived to 85–90% of their free feeding weight to increase spontaneous exploration, except during recovery from surgery, where food was available *ad libitum*. The water remained available *ad libitum* throughout the study. All experimentation was conducted in accordance with the Institutional Animal Care and Use Committee of the Favaloro University.

### 2.2. Surgery and cannulation

All rats that were used for pharmacological infusions were implanted bilaterally in Prh with 22-gauge indwelling guide cannulas. Subjects were anesthetized with ketamine (Holliday, 74 mg/kg, i.p.) and xylazine (Konig, 7.4 mg/kg, i.p.) and placed in a stereotaxic frame (David Kopf Instruments, Tujunga, CA, USA) with the incisor bar set at −3.2 mm. Guide cannulas were implanted according to the following coordinates, measured relative to the skull at bregma (Paxinos and Watson, 1998) anteroposterior −5.5 mm, lateral ± 6.6 mm, dorsoventral −7.1 mm. The cannulas were secured to the skull using dental acrylic. Obturators, cut to sit flush with the tip of the guide cannulas and with an outer diameter of 0.36 mm, were inserted into the guides and remained there until the first infusions. At the completion of each surgery, an antibiotic was applied for 3 days (Enrofloxacin; 0.27 mg kg-1, Vetanco, Arg). Animals were given approximately 7 days to recover before behavioral testing and drug infusions.

### 2.3. Infusion procedure

A Tat-conjugated peptide, designed to block the binding of dynamin to amphiphysin and thus prevent endocytosis (Gout et al., 1993; Lissin et al., 1998; Kittler et al., 2000; Lin et al., 2011), was infused in order to block endocytosis. Depending on the experiment, rats received bilateral infusions of Tat P4 peptide and Tat Scrambled control peptide (S) (60μg/μl/0.5 μl side; Cambridge, UK), human recombinant BDNF or saline (0.5 μg/μl/0.5 μl side), ANA-12 or saline at different times during the behavioral task. The injection volume was always 0.5 μl/side. Sequences are as follows: amino acid sequence for the dynamin inhibitory peptide (P4) is QVPSRPNRAP, and for the Scrambled control peptide (S) is QPPASNPRVR.

Bilateral infusions were conducted simultaneously using two 5-μl Hamilton syringes that were connected to the infusion cannulas by propylene tubing. Syringes were driven by a Harvard Apparatus precision syringe pump, which delivered 0.5 μl to each hemisphere over 1 min. The infusion cannulas were left in place for an additional minute to allow for diffusion. At least 3 days were allowed for washout between repeated infusions.

### 2.4. Apparatus

For the behavioral procedures, an open triangular acrylic field was used as an arena, each wall 60 cm long by 60 cm high. The walls of the triangular open field were higher to minimize the visual access to the distal cues in the room. The arena was located in the middle of a room with dim lighting, and the floor was always covered with wood shavings. A video camera was positioned on the arena in order to record both the sample session and the evaluation session for later analysis. The objects to be used were created by attaching together two small objects, depending on the condition to be studied, similar or dissimilar. Different objects were used for our within-subject design, all of them made of different materials, such as metal, glass or plastic. All of the object were approximately between 8 and 15 centimeters tall, and 4 to 7 centimeters width. All objects were adhered to the open field floor with reusable adhesive putty and cleaned with 50% ethanol solution between sessions, both sample and choice phases. For the task, the three composite objects were aligned closely to one of the arena walls and the position of each object was counterbalanced.

### 2.5. Behavioral procedures

For the Spontaneous Object Recognition (SOR) task (Figure 1A), each rat was handled for 3 days and then habituated to the arena for 5 min per day for 3 days before exposure to the objects. After habituation, the rats were exposed during a 5 min duration sample phase to three objects made of two features depending on the condition. For the similar condition, two of the objects shared one feature (AB and BC) and the third object was made of two other different features (EF). For the dissimilar condition, all three objects were made of different features (AB, CD, and EF). Twenty-four hours after sample phase, we conduct a choice phase, of 3 min duration, in which the animals were exposed to two objects, a novel one and a familiar one, and depending on the condition evaluated, the objects varied in composition. For the similar condition, the novel object was made of the two non-shared features of the objects presented in the sample phase (AC), and the familiar object was a copy of the third object (EF). For the dissimilar condition, the novel object was made of two novel features (KI) and the familiar object was a copy of the object presented during the sample phase (AB, CD, and EF).

For the extra-similar condition, the process was the same as the similar condition, differing only in the objects used. During sample phase, animals were exposed during 5 min to three different objects, and two of those shared one feature (ABB and BBC), while the third object was made of different features (EFG). During the choice phase, 24 h later, the animals were exposed to a novel object was made of a novel combination of familiar features (ABC), and the familiar object was a copy of the third object presented in the sample phase (EFG) (Figure 3D). Exploration was recorded and later scored manually for both the sample and choice phases. For all experiments, exploration of a particular object was defined as the rat having its nose directed at the object at 2 cm or less or touching the object with its nose. Rearing with the head oriented upward did not count as exploration. Climbing over or sitting on the object was not included.

In every trial, objects were pseudorandomly assigned to a different location in the arena to avoid a bias for locations within the arena.

### 2.6. Statistical analysis

For all the experiments, the results were expressed as a discrimination ratio calculated as the time exploring the novel object minus the time exploring the familiar object divided by the total exploration time [(tnovel-tfamiliar)/(ttotal)].

For the sample phases, the percentage of time spent exploring each object was compared using a repeated-measures two-way ANOVA or one-way ANOVA. For the choice phases, we performed one-sample *t*-test for every discrimination ratio to analyze whether control animals learned the task, verifying that the ratio was different from zero. Discrimination ratios were compared within subject using a paired *t*-test, one way ANOVA or two-way ANOVA.

Some experiment has female and male animals, and the statistical analyses was made with pooled data, except for the first experiment (Figure 1). The experiments in which there are animals of both sexes, the number of animals of each sex is detailed in the results.

## 3. Results

### 3.1. Female rats, as well as male rats, can spontaneously store and disambiguate the representations of similar and dissimilar objects

It has already been shown that object exploration and preference is driven by novelty in the modified version of the SOR task in male rats (Miranda et al., 2017). This task includes a similar and a dissimilar condition in which the load on pattern separation is different (Figure 1A; see M and M). The first goal of this work was to test the performance of female rats in the same SOR task version and compare the result with male rats (Figure 1).

Six male Long Evans and 6 female Long Evans were used for this experiment, all animals underwent both the similar (s-SOR) and dissimilar (d-SOR) conditions. We did not find a difference in the percentage of time the animals spend exploring the objects during the sample phase {repeated measures one way ANOVA: females s-SOR [F (1.588, 7.942) = 1.829, *p* = 0.2211]}; males s-SOR [F (1.147, 5.734) = 3.364, *p* = 0.1171]; females d-SOR [F (1.457, 7.287) = 3.202, *p* = 0.1081]; males d-SOR [F (1.438, 7.190) = 2.240, *p* = 0.1779)]. There was no significant interaction between the objects by condition and sex (repeated measures two-way ANOVA: psex = 0.6109, pcondition + object = 0.0745, pinteraction = 0.2793).

Also, we did not find any differences in total exploration times (female s-SOR = 39.78 ± 5.09, male s-SOR = 39.63 ± 5.21, female d-SOR = 30.08 ± 3.94, male d-SOR = 36.88 ± 1.92) comparing sexes in the same condition (paired *t*-test: female versus male similar, *p* = 0.9840; female versus male dissimilar, *p* = 0.1518).

The choice phase was conducted 24 h after the sample phase for both conditions and memory was evaluated by comparing the amount of time spent exploring a novel object and familiar object. This comparison was expressed in the discrimination ratio. There were no significant differences between the discrimination ratio for both sexes in the similar and the dissimilar condition (pconditions = 0.0819, psex = 0.6047, pinteraction = 0.4822) (Figure 1B, Supplementary Table 1). Also, we did not find any differences in total exploration times comparing sexes in the same condition (paired *t*-test: female versus male similar, *p* = 0.9840; female versus male dissimilar, *p* = 0.1518) (Table 1). One-sample *t*-tests against a value of zero indicated that both sexes were able to learn the task in both conditions [One-sample *t*-test (s-SOR females, *t* = 6.146), *p* = 0.0017; one- sample *t*-test (s-SOR males, *t* = 6.740), *p* = 0.0011; d-SOR females, *t* = 3.277), *p* = 0.0220; one- sample *t*-test (d-SOR males, *t* = 5.128, *p* = 0.0037)].

**TABLE 1:** Total exploration times during the choice session of the SOR task.

Figure	*P*-value	T total
1C	0.9840	
s-SOR female		39.78 ± 5.09
s-SOR male		39.63 ± 5.21
	0.1518	
d-SOR female		30.08 ± 3.94
d-SOR male		36.88 ± 1.92
2C	0.3567	
s-SOR Tat-s		35.66 ± 6.05
s-SOR Tat-P4		29.52 ± 4.32
2F	0.1128	
d-SOR Tat-s		45.42 ± 3.84
d-SOR Tat-P4		36.77 ± 3.83
3C	0.0792	
s-SOR Tat-S		32.17 ± 2.83
s-SOR Tat-P4		27.04 ± 2.24
**3F**		
Tat-S/Vehicle		57.57 ± 4.97
Tat-S/hrBDNF		53.36 ± 5.06
Tat-P4/hrBDNF		49.58 ± 7.34
4C	0.8451	
Vehícle		25.68 ± 1.25
ANA-12		26.15 ± 2.06
4F	0.8111	
Vehicle		38.44 ± 3.70
ANA-12		37.57 ± 1.84
5C	0.6272	
Unilateral		46.84 ± 3.93
Contralateral		44.15 ± 1.88

Results are expressed as mean ± SEM in seconds. *P*-values are for the comparison between total exploration times during the choice session for each experimental group depicted in the same row. Paired *t*-test was used for these comparisons, except in the case of Figure 3F, for which one-way ANOVA was used.

These results indicate that intact female rats were able to spontaneously disambiguate the representations of two similar object seen 24 h before, and that there is no difference between sexes for performance in this specific task.

### 3.2. Endocytosis in the Prh is required for consolidation of similar, but not for dissimilar object memory representations

We then proceeded to study the role of the endocytosis in the formation of differentiated representations in the Prh in male and female rats. If mechanisms of receptor internalization are specifically required in the Prh for memory differentiation, then blocking internalization should alter the similar version of the SOR task, without affecting the dissimilar version. To test this hypothesis, we blocked the putative receptor internalization immediately after the sample phase. We used a Tat-conjugated peptide (Tat-P4) and a scrambled control peptide (Tat-S) design to prevent endocytosis by blocking the binding of dynamin (Gout et al., 1993; Lissin et al., 1998; Lin et al., 2011). Tat-P4 or Tat-S was injected in Prh immediately after the sample phase, and the memory of animals was test 24 h later in both conditions, similar (*n* = 12, 6 male and 6 female) and dissimilar (*n* = 11, 5 male and 6 female) (Figures 2A, D). Animals from both sexes were pooled and analyzed altogether, and all animals underwent the experimental (Tat-P4) and the control (Tat-S) conditions. A discrimination index above zero indicates a significant discrimination and a reasonable memory retention. One-sample *t*-tests against a value of zero indicated that Tat-S injected animals were able to learn both the s-SOR and the d-SOR [One-sample *t*-test (d-SOR Tat-S, *t* = 7.735), *p* < 0.001; one- sample *t*-test (s-SOR Tat-S, *t* = 3.071), *p* = 0.0106] (Figures 2C, F), whereas Tat-P4 injected animals only learned the d-SOR version [One-sample *t*-test (d-SOR Tat-P4, *t* = 4.655), *p* = 0.0009; one- sample *t*-test (s-SOR Tat-P4, *t* = 1.142), *p* = 0.2776)] (Figures 2C, F). We found a significant difference between Tat-S and Tat-P4 injected animals in the choice phase in the s-SOR [Tat-S vs. Tat-P4 paired *t*-test (*t* = 2.899), *p* = 0.0145, *n* = 12] (Figure 2C). There were no differences in total exploration times between groups (see Table 1). These results indicate that endocytosis is important to spontaneously disambiguate the memory representations of two similar objects.

### 3.3. Endocytosis is required in a time-restricted windows for consolidation of similar object memory representations

Memory consolidation is a process that occurs during a restricted time window (McGaugh, 2000; Winters and Bussey, 2005b). To test whether Tat-P4 interfered with memory during a restricted delay after the sample phase, Tat-P4 or Tat-S was injected into the Prh 5 h after the sample phase, and rats were tested 24 h later, all animals underwent both drug conditions (*n* = 7, 2 males, 5 female) (Figure 3A). Since in the previous experiment we found an effect only in the similar condition, we decided to test this time window in this specific condition. Injection of the TatP4 did not change total exploration times compared with Tat-S (see Table 1). We did not observe any memory impairments in the s-SOR when Tat-P4 was injected 5 h after sampling the objects [One-sample *t*-test (Tat-S, *t* = 13.88), *p* < 0.0001; one- sample *t*-test (Tat-P4, *t* = 7.377), *p* = 0.0003], indicating that Tat-P4 injected animals were able to learn the similar condition as successfully as Tat-S-injected animals [Tat-S vs. Tat-P4 paired *t*-test (*t* = 0.2985), *p* = 0.7754, *n* = 7] (Figure 3C). In sum, this result indicates that extending the time interval between sample phase and Tat-P4 infusion reduces the disrupting effect of the peptide on the choice phase.

### 3.4. Is BDNF acting through endocytosis to promote differentiation discrimination?

Brain derived neurotrophic factor enhances memory consolidation in several tasks if injected exogenously (Alonso et al., 2002; Peters et al., 2010; Bekinschtein et al., 2013). We wondered if the enhancing effect found in previous studies could be prevented if we blocked the internalization of receptors. We have previously shown that BDNF in Prh is critical for this task. In particular, injection of exogenous BDNF into Prh enhanced discrimination of similar object memories (Miranda et al., 2017). To be able to see any memory improvement induced by BDNF in pattern separation, we used a slightly different version of the SOR task, the extra-similar SOR (xs-SOR) (Miranda et al., 2017) in which we make discrimination more difficult by bringing the performance of the control animals down. The key modification to the task is making the objects more similar during the sample phase. In this particular experiment, we used three groups of rats, a group injected in Prh with Tat-S and saline 15 min prior to sample phase, a second group injected with Tat-S 15 min prior and human recombinant BDNF (hrBDNF) immediately after the sample phase, and a third group injected with Tat-P4 15 min prior and hrBDNF immediately after sample phase. All animals were exposed to the three treatments (*n* = 12, all males) (Figure 3D).

There were no differences in exploration of the three objects during the sample phase (repeated-measures two-way ANOVA, xs-SOR: *F* = 1.000, pdrug = 0.1748; *F* = 0.1089 pobjects = 0.8668, *F* = 1.191, pinteraction = 0.3282) (Figure 3E). A one-way between-subjects ANOVA was conducted to compare the effect of the treatments in the choice phase. There was a significant difference between treatments at the *p* < 0.05 level [*F* (Rolls and Kesner, 2006; Miranda et al., 2017) = 3.838, *p* = 0.043]. *Post hoc* comparison using Tukey’s multiple comparison test indicated that the mean score for the Tat-S/Veh condition (*M* = −0.02, SEM = 0.037) was significantly different than the Tat-S/BDNF condition (*M* = 0.11, SEM = 0.036) and different than the Tat-P4/BDNF condition (*M* = 0.08, SEM = 0.038). However, the Tat-S/BDNF condition did not significantly differ from the Tat-P4/BDNF condition (Figure 3F). This experiment was inconclusive, as it appears that BDNF was not able to improve discrimination in Tat-P4-injected animals, but the discrimination index did not differ from that of the Tat-S/BDNF group.

### 3.5. Interaction between BDNF–TrkB signaling and endocytosis in pattern separation

**FIGURE 4:**
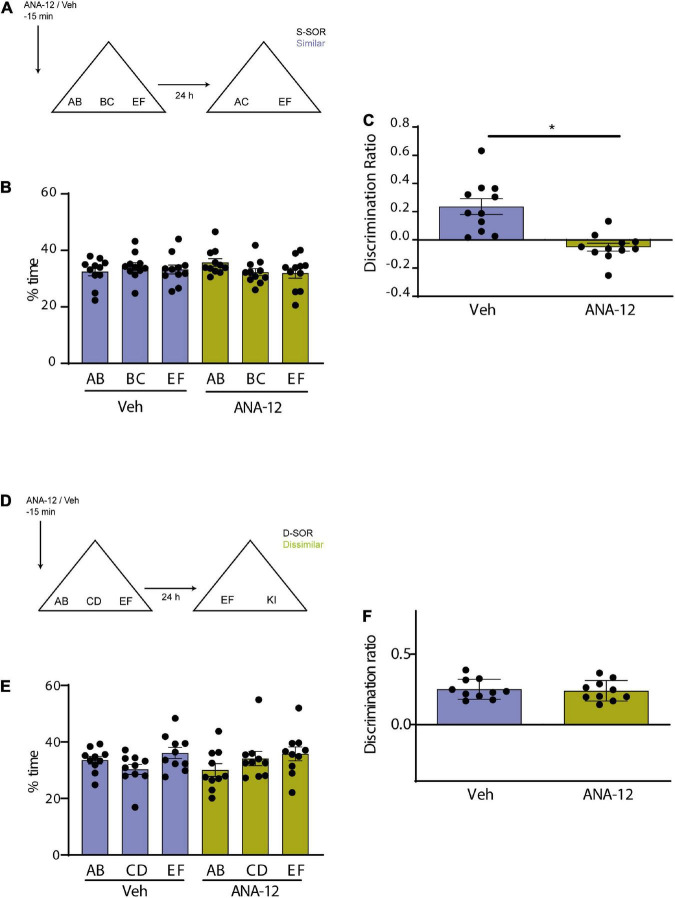
**(A)** Schematic illustration of the similar-spontaneous object recognition task (s-SOR) task indicating when ANA-12/Veh was infused 15 min before the sample phase. **(B)** Percentage of time spent exploring each of the objects in the sample phase. Repeated-measures two-way ANOVA (%time) *F* = 1.000, pdrug = 0.3409, *F* = 0.3029, pobject = 0.7420, *F* = 1.342, pinteraction = 0.2839. **(C)** Effect of ANA-12 or Veh injections on the discrimination ratios for the s-SOR version of the task. Paired *t*-test, *p* = 0.0005, *t* = 4.995, *n* = 11. **(D)** Schematic illustration of the d-SOR task indicating when ANA-12/Veh was infused 15 min before the sample phase. **(E)** Percentage of time spent exploring each of the objects in the sample phase. Repeated-measures two-way ANOVA (%time) *F* = 1.976, pdrug = 0.1934, *F* = 2.066, pobject = 0.1557, *F* = 0.8339, pinteraction = 0.4504. **(F)** Effect of ANA-12 or Veh injections on the discrimination ratios for the d-SOR version of the task. Paired *t*-test, *p* = 0.0005, *t* = 4.995, *n* = 10. *p < 0.05.

We wanted to use a different strategy to evaluate the possible interaction between BDNF and endocytosis by blocking both the BDNF receptor TrkB and endocytosis in a molecular disconnection experiment (Miranda et al., 2017). We first tested the effect of Prh injection of ANA-12, a selective non-competitive antagonist of TrkB, BDNF receptor (Cazorla et al., 2011). We injected all animals with ANA-12 or saline in Prh 15 min prior to the sample phase and evaluated memory 24 h after training in both similar and dissimilar version (Figures 4A, D). There were no differences in total exploration times in neither the similar (*n* = 11, all female) nor the dissimilar (*n* = 10, all female) version of the task (paired *t*-test, similar, *p* = 0.6742; dissimilar, *p* = 0.8804) (Table 2). We did not find any interactions between drugs or objects (repeated-measures two-way ANOVA, d-SOR: pdrug = 0.1934, pobjects = 0.1557, pinteraction = 0.4504; s-SOR: pdrug = 0.3409, pobjects = 0.7420, pinteraction = 0.2839) (Figures 4B, E). We observed a memory impairment in the s-SOR version of the task (paired *t*-test, *p* = 0.0005, *t* = 4.995) (Figure 4C), but not in the d-SOR version (paired *t*-test, *p* = 0.7462, *t* = 0.3337) (Figure 4F), Thus, blocking TrkB only generated a deficit in the “similar” condition, disabling animal’s capacity of discrimination of overlapping memories.

**TABLE 2:** Total exploration times during the sample session of the spontaneous object recognition (SOR) task.

Figure	*P*-value	T total
2B	0.3321	
s-SOR Tat-s		81.70 ± 8.71
s-SOR Tat-P4		90.58 ± 9.33
2E	0.9826	
d-SOR Tat-s		75.06 ± 6.43
d-SOR Tat-P4		74.76 ± 10.61
3B	0.3453	
s-SOR Tat-S		82.74 ± 4.63
s-SOR Tat-P4		74.30 ± 5.65
**3E**		
Tat-S/Vehicle		125.9 ± 10.55
Tat-S/hrBDNF		126.4 ± 11.7
Tat-P4/hrBDNF		117.7 ± 14.6
4B	0.4431	
Vehícle		61.27 ± 6.76
ANA-12		54.15 ± 4.53
4E	0.8804	
Vehicle		64.45 ± 3.61
ANA-12		63.52 ± 4.68
5B	0.5426	
Unilateral		91.72 ± 10.18
Contralateral		85.97 ± 6.09

Results are expressed as mean ± SEM in seconds.

**FIGURE 5:**
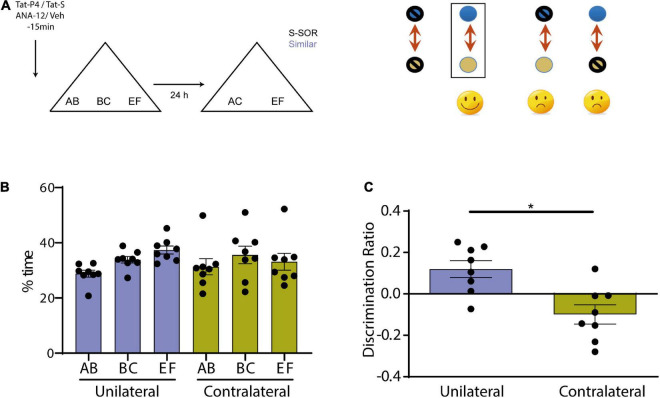
**(A)** Schematic illustration of the similar-spontaneous object recognition task (s-SOR) task indicating when ANA-12/TatS and Veh/TatP4 was infused (left). In a molecular disconnection experiment, there is an inactivation of two elements of a signal transduction pathway either in the same hemisphere or in both of them. While in the first case, the putative pathway that links both proteins remain intact and functional in one hemisphere, the inactivation of one element in one hemisphere and the other element in the other hemisphere prevents functionality of the putative interaction pathway in both hemispheres (right). **(B)** Percentage of time spent exploring each of the objects in the sample phase. Repeated-measures two-way ANOVA (%time), s-SOR: pcondition = 0.7318, pobject = 0.1943, pinteraction = 0.4355. **(C)** We found no effect in the similar SOR task when ANA-12 and TatP4 were injected in the same hemisphere [One-sample *t*-test (same, *t* = 2.964), *p* = 0.0210]. However, when ANA-12/TatS and Veh/TatP4 where injected in different hemispheres in Prh, we found a significant impairment in the similar SOR task [One-sample *t*-test (different, *t* = 2.150), *p* = 0.0686; paired *t*-test same vs. different, *p* = 0.0090, *t* = 3.574, *n* = 8]. *p < 0.05.

We next evaluated whether BDNF pathway and endocytosis interacted during consolidation of similar object memories in the SOR task. We used a protocol of molecular disconnection that we have carried out in previous studies (Miranda et al., 2017). The logic underlying this is the same that in any brain disconnection experiment that tries to determinate if, during a specific behavioral manipulation, two brain structures are connected (Gaffan et al., 1989; Ito et al., 2008). If we assume that the principal connection between two structures is in the same hemisphere (ipsilateral), the deactivation or lesion of the two regions in the same side will keep the behavior intact, but the contralateral deactivation will affect the performance. If we consider two molecules or gene expression pathways in specific given structure instead of two regions, a similar method of reasoning can be used. If two molecular pathways interact to produce a specific behavior, blocking both pathways in that area of only one hemisphere will have no effect; but if one pathway is blocked in one hemisphere and the second pathway in the other, we would see a deficit. Thus, we evaluated if BDNF and endocytosis interacted in Prh during consolidation of similar memories by blocking both pathways in the same hemisphere or blocking BDNF in one hemisphere and endocytosis in the other. We injected ANA-12/Veh or Tat-P4/Tat-S in Prh 15 min before the sample phase and evaluated memory 24 h after it (*n* = 8, all female) (Figure 5A). All animals underwent both treatment conditions. There were no differences in total exploration time between the two groups (paired *t*-test, *p* = 0.5426, *t* = 0.6399, *n* = 8), nor between objects (repeated-measures two-way ANOVA, s-SOR: pcondition = 0.7318, pobject = 0.1943, pinteraction = 0.4355) (Figure 5B).

We found no effect in the similar SOR task when ANA-12 and TatP4 were injected in the same hemisphere (and their corresponding vehicles were injected in the other hemisphere) (Figure 5C), however, when ANA-12/TatS and Veh/TatP4 where injected in different hemispheres in Prh, we found a significant impairment in the similar SOR task (Figure 5C) (paired *t*-test unilateral vs. contralateral, *p* = 0.0090, *t* = 3.574). There were no differences in total exploration times between the two groups (see Table 1). One sample *t*-test showed that the discrimination ratio from the “unilateral” group was different from zero, whereas the discrimination ratio from the “contralateral” group was not (p_unilateral_ = 0.0210, *t* = 2.96; *p*_contralateral_ = 0.0686, *t* = 2.150). This result suggests that BDNF and endocytosis interacts during consolidation of similar memories in Prh.

## 4. Discussion

The key finding of this study are: (1) blocking endocytosis in Prh impairs consolidation of similar, but not dissimilar object memories, (2) blocking BDNF TrkB receptors prevents consolidation of similar objects in Prh, (3) an interaction between endocytosis and BDNF is necessary for appropriate memory differentiation, and (4) we found no sex differences in this particular task. In accordance with previous studies that have shown the importance of the different receptors in memory consolidation (Winters and Bussey, 2005a; Banks et al., 2014; Sanchez-Mejias et al., 2020), we contribute with evidence that shows that endocytosis in general affects memory consolidation of similar representations during an object recognition task in Prh.

In this study, we hypothesize that impairing receptor trafficking interferes with the plasticity of Prh, disturbing memory consolidation. This is consistent with previous findings showing that blocking receptor trafficking impairs object recognition memory. For example, the blockade of NMDARs after the sample phase impaired both object recognition memory (Winters and Bussey, 2005a) and long- term potentiation (LTP) (Barker et al., 2006). A study using a Tat-conjugated peptide to block the endocytosis of AMPAR also impaired object recognition memory, but only when injected prior to the retrieval phase (Cazakoff and Howland, 2011), while a short-term treatment with Flumazenil, a GABAr antagonist, improved long term memory in the Novel Object Recognition task in a mouse model of Down’s Syndrome, which is characterized by a cognitive deficit generated by excessive neuronal inhibitory tone (Colas et al., 2017). In this study, we bounce into endocytosis-dependent trafficking, impairing object recognition memory. Endocytosis could act to facilitate memory differentiation by different mechanisms: (1) AMPA receptor internalization could be leading to a greater malleability of plasticity mechanism or (2) GABA receptor endocytosis could decrease postsynaptic inhibition, hence facilitating synaptic plasticity. If indeed it were so, the interference in the mechanisms of internalization of receptors would lead to a non-discrimination behavior when the pattern separation load is high but not when it is low. Due to the fact that we used a general method to impair endocytosis, we cannot establish the trafficking of which particular receptors was affected.

Tat-P4 is a dynamin inhibitory peptide, it has been designed to block the binding of dynamin to amphiphysin, and thus it prevents endocytosis. Blockade of the endocytic pathway by this peptide has been more thoroughly studied *in vitro* (Zheng et al., 2008; Cosker and Segal, 2014; Moya-Alvarado et al., 2018; González-Gutiérrez et al., 2020). We did not perform a thorough time course analysis of the Tat-P4 peptide blocking action. In a study involving fear-potentiated startle and infusion of Tat-P4 in the amygdala, the authors found a strong behavioral (impaired reinstatement) and molecular effect (blockade of GABAr endocytosis) when the peptide was injected 30 min before testing (Cosker and Segal, 2014). In a study using cultured cells, a plateau on GABA-A mIPSC increase was found around 40 to 50 min after treatment with the Tat-P4 peptide (Lin et al., 2011). In our experiments, we only found an effect on behavior when Tat-P4 was injected either 15 min before the sample phase or immediately after, but not 5 h after. This is consistent with previous studies showing that the blocking action of the peptide begins within minutes (Zheng et al., 2008; Cosker and Segal, 2014; Moya-Alvarado et al., 2018; González-Gutiérrez et al., 2020).

Changes in synaptic strength are thought to support long-term memory in the brain (Kandel, 2001). It has been proposed that LTP and long-term depression (LTD) are key processes underlying memory storage in several different neural regions (Bliss and Collingridge, 1993; Martin et al., 2000; Kandel, 2001). In particular, both of these forms of synaptic plasticity have been found in Prh (Bilkey, 1996; Ziakopoulos et al., 1999; Cho et al., 2000; Massey et al., 2001), Nevertheless, object recognition memory has been strongly linked with the induction and maintenance of LTD in this particular structure [see review (Miranda and Bekinschtein, 2018)]. Both LTP and LTD involve AMPAR and NMDAR-dependent mechanisms in the Prh (Bilkey, 1996; Ziakopoulos et al., 1999). It has been shown that NMDAR-dependent LTD requires the internalization of AMPA receptors in Prh (Griffiths et al., 2008), while LTP is associated with perirhinal NMDAR (Barker et al., 2006) and was shown to also recruit GABA-dependent mechanisms in this structure (Kotak et al., 2017). While there are currently many results that point at LTD as the key mechanism of synaptic plasticity for object recognition in the Prh, it is possible that a balance between LTD and LTP is needed for the maintenance of consolidation and storage of distinguished representations of object memories (Miranda and Bekinschtein, 2018).

Brain derived neurotrophic factor is considered to be an important part of the cellular mechanism that supports the formation and maintenance of memory by promoting synaptic consolidation. Accordingly, BDNF also generates changes in spine shape, leading to the stabilization of LTP and, as a result, increased memory storage (Bramham and Messaoudi, 2005). Previous studies have also shown the importance of BDNF in object recognition memory (Seoane et al., 2011). In our studies, we found that blocking the expression BDNF in Prh using a BDNF antisense oligonucleotide impaired only the performance in the similar condition of the SOR task, showing the existence of a specific mechanisms underlying storage of unique representations of objects in Prh (Miranda et al., 2017). In this particular study, we did not block the expression of BDNF, instead we used a not competitive antagonist (ANA-12) to prevent the activation of BDNF receptor, TrkB, obtaining similar results. Inhibiting TrkB impaired memory consolidation of similar but not distinct objects. We also evaluated if BDNF and endocytosis signaling pathways are connected in Prh using a molecular disconnection experiment. The result suggests that BDNF and endocytosis interact during consolidation of overlapping memories in Prh. However, we also tested if human recombinant BDNF could enhance object recognition memory in an endocytosis-dependent manner. We predicted that the enhancing effect of BDNF would be prevented when we blocked the trafficking receptor. We found that BDNF did enhance the consolidation of extra-similar memories, but we did not find a significant effect of blocking endocytosis using Tat-P4. However, animals injected with BDNF and Tat-P4 seem to remember worse than BDNF control animals. There are a number of reasons why this experiment was not conclusive. For example, exogenous BDNF might engage a different mechanism than that of physiological BDNF in which endocytosis is partially required. In addition, the dose of Tat-P4 peptide could not have been enough to block the effect of a large exogenous BDNF dose. Our current data is not sufficient to make a conclusion form this particular experiment. Nevertheless, the robustness of the molecular disconnection result strongly suggests that there is an interaction between BDNF and endocytosis in the Prh during consolidation of similar overlapping object memories.

In conclusion, our experiments suggest that BDNF and endocytosis are essential for consolidation of separate memories and a part of a time-restricted, protein synthesis-dependent mechanism of memory stabilization in Prh during storage of object representations. These results agree with previous investigation that showed the critical importance of BDNF for this type of memories and the molecular mechanisms underlying this process (Miranda et al., 2017).

To our knowledge, the present study is the first to provide evidence regarding the role of endocytosis in the consolidation of overlapping memories in the Prh and the first to test this task in female rats. Together with previous studies, we reinforce the importance that BDNF as a plasticity molecule involved in this process across different brain regions.

## Data availability statement

The raw data supporting the conclusions of this article will be made available by the authors, without undue reservation.

## Ethics statement

All experimentation was conducted in accordance with the Institutional Animal Care and Use Committee of the Favaloro University, Buenos Aires, Argentina.

## Author contributions

DP was responsible for all the experiments and drafting the manuscript. MM contributed to the experiments. MG was responsible for critically revising and correcting the manuscript. PB was responsible for the general idea and critically revising and correcting the manuscript. NW contributed to the general idea and final revision of the manuscript. All authors read and approved the final manuscript and contributed to the conception of the work.
