# Supplementary material for: Endocytosis is required for consolidation of pattern-separated memories in the perirhinal cortex
Source: Front Syst Neurosci. 2023 Feb 23;17:1043664. doi: 10.3389/fnsys.2023.1043664 (PMC9995888; doi:10.3389/fnsys.2023.1043664)
Supplement: Supplementary file 1 [file Table_1.docx]

Supplementary Table 1. Total exploration times during choice session of the SOR task.

| Figure | Novel | Familiar |
| --- | --- | --- |
| 1C |  |  |
| s-SOR male | 23,25 ± 3,05 | 17,22 ± 2,06 |
| s-SOR female | 22,52 ± 2,92 | 17,27 ± 2,21 |
| d-SOR male | 20,77 ± 0.41 | 14,12 ± 0.51 |
| d-SOR female | 17,43 ± 2,02 | 10,98 ± 1,57 |
| 2C |  |  |
| s-SOR Tat-s | 20,94 ± 3,63 | 14,72 ± 2,57 |
| s-SOR Tat-P4 | 13,88 ± 2,32 | 15,65 ± 2,34 |
| 2F |  |  |
| d-SOR Tat-s | 26,81 ± 2,36 | 18,61 ± 1,6 |
| d-SOR Tat-P4 | 21,09 ± 1,77 | 15,68 ± 2,37 |
| 3C |  |  |
| s-SOR Tat-S | 20,10 ± 1,61 | 12,07 ± 1,25 |
| s-SOR Tat-P4 | 18,11 ± 1,83 | 8,92 ± 1,1 |
| 3F |  |  |
| Tat-S / Vehicle | 28,05 ± 3,34 | 28,65 ± 2,42 |
| Tat-S / hrBDNF | 30,12 ± 3,15 | 23,47 ± 2,66 |
| Tat-P4 / hrBDNF | 28,65 ± 3,57 | 25,06 ± 3,44 |
| 4C |  |  |
| Vehícle | 16,17 ± 1,20 | 9,86 ± 0,73 |
| ANA-12 | 12,58 ± 1,23 | 13,57 ± 0,89 |
| 4F |  |  |
| Vehicle | 24,08 ± 2,34 | 14,36 ± 1,49 |
| ANA-12 | 23,17 ± 0,94 | 14,40 ± 1,009 |
| 5C |  |  |
| Unilateral | 26,39 ± 2,76 | 20,45 ± 1,58 |
| Contralateral | 19,99 ± 1,53 | 24,16 ± 1,08 |

Results are expressed as mean ± SEM in seconds.
